# 

**Published:** 1952-04

**Authors:** 


### THE BRISTOL MEDICO-CHIRURGICAL JOURNAL

*A Journal of the Medical Sciences for the*

WEST OF ENGLAND AND SOUTH WALES

VOL. 69 APRIL 1952 NO. 250

*PUBLISHED QUARTERLY*

*ANNUAL SUBSCRIPTION 16/- POST FREE*

PUBLISHED UNDER THE AUSPICES OF THE
BRISTOL MEDICO-CHIRURGICAL SOCIETY

### Editorial Committee

MR. H. KEITH LUCAS, 60 St. John's Road, Bristol 8. *Editor.*
DR. J. E. CATES, The Dept. of Medicine, Bristol Royal Infirmary. *Assistant Editor.*
DR. G. E. F. Sutton, 1 Pembroke Road, Bristol 8. *Treasurer.*
DR. J. H. MIDDLEMISS, 11 Pembroke Road, Bristol 8.
DR. A. M. MACLACHLAN, 167 Redland Road, Bristol 6.
DR. A. H. GALE, The University, Bristol 8.

*Local Correspondents*

*Bath* . . Mr. A. D. BATEMAN, 3 The Circus, Bath.
*Exeter* . . Dr. L. N. JACKSON, Mount Jocelyn, Crediton, Devon.
*Gloucester* . Dr. R. F. JARRETT, 9 College Green, Gloucester.
*Newport* . Mr. J. L. D. WILLIAMS, The Knoll, 145 Stow Hill, Newport, Mon.
*Plymouth* . Dr. C. R. CROFT, 1 The Crescent, Hartley, near Plymouth.
*Taunton* . Dr. M. MCALLEY, 1 The Crescent, Taunton.
*Torquay* . Dr. A. ROBINSON THOMAS, 20 Devon Square, Newton Abbot, Devon.
*Truro* . . Dr. F. D. M. HOCKING, St. Andrews, Perranporth, Cornwall.
*Yeovil* . . Mr. J. A. VERE NICHOLL, Knapp House, Yeovil, Somerset.

### NOTICE TO CONTRIBUTORS

Original articles are invited, provided they have not been published elsewhere. Notes of forthcoming events, meetings held, degrees conferred, staff appointments, and other local news are welcomed. The editorial committee reserves the right to make literary corrections

Illustrations should always be supplied ready for reproduction. All re-touchings or other operations required will be charged to the author. Line drawings should be drawn in Indian ink. We regret that we must ask authors to pay for making blocks, if this is necessary.

Authors of original articles are entitled to receive twenty-five Reprints, with covers showing source of Reprint, free of charge. Additional reprints may be obtained if notice is given at the time of returning proofs. Approximate cost, with cover as above: 8 pp., 25 copies, **8/-**; 50 copies, **12/6**. Contributors requiring special covers for their reprints, showing source of reprint with author's name, appointments and title of article, can obtain them as follows: 25 copies, **12/-**; 50 copies, **14/6**.

J. W. ARROWSMITH LTD., SMALL STREET,
BRISTOL.

### *Appointments*

*Bristol*

Aron, K. W., M.B., D.P.M.: Senior Registrar in Psychiatry, Bristol Mental Hospital.

Bodenham, D. C., M.B., F.R.C.S.: Consultant Plastic Surgeon to Out-Patients, United Bristol Hospitals.

Copland, W. A., M.B.: Registrar in Radiology (Diagnostic), United Bristol Hospitals.

Inman, G. K. E., M.B., D.M.R.D.: Senior Registrar in Radiology (Diagnostic), Southmead and Frenchay Hospitals.

Jaco, N. T., B.M., M.R.C.P., D.C.H.: Registrar in Paediatrics, Southmead Hospital.

Logan, P., M.D., M.R.C.P.I.: Registrar in Diseases of Chest, Ham Green Hospital.

March, H. P., M.R.C.P., D.M.R.D.: Senior Registrar in Radiology, United Bristol Hospitals.

Monks, P. J. W., M.B., F.R.C.S.: Registrar in General Surgery, Frenchay and Southmead Hospitals.

Nicol, W. A., L.D.S., D.D.O.: Assistant Orthodontist, Bristol Dental Hospital.

Parkinson, T. J., M.B., D.C.H.: Registrar in General Medicine, Southmead Hospital.

Schooling, I. B., M.B., D.C.H.: Registrar in Anaesthetics, United Bristol Hospitals.

Turner, W. D., M.B.: Registrar in Anaesthetics, United Bristol Hospitals.

*Gloucestershire*

Ferguson, H., M.B., D.M.R.D.: Senior Registrar in Radiology, North Gloucestershire.

*Plymouth*

Cary Lynch, G. A., D.P.H.: Consultant Pathologist, Plymouth.

Forbes, H. A. W., B.M., M.R.C.P.: Consultant Physician, Plymouth.

Vellacott, H. D. S., M.B., F.R.C.S.: Consultant Surgeon, Plymouth.

*New and Recent Books*

**MODERN TREATMENT YEARBOOK 1952**

EIGHTEENTH YEAR OF ISSUE

A yearbook of diagnosis and treatment edited by SIR CECIL WAKELEY, K.B.E., C.B., M.CH., F.R.C.S., F.R.S.E., F.A.C.S., "A very serviceable and particularly useful presentation of modern methods."—*British Medical Journal.*
Pp. viii + 360, with 31 plates. *(Postage 1s., abroad 2s.)* 17s. 6d.

**SURGICAL TREATMENT OF FACIAL INJURIES**

By V. H. KANZANJIAN, M.D., D.M.D., and J. M. CONVERSE, M.D. "A fine book . . . could be studied with advantage by all concerned with this type of work."—*The Lancet.*
Pp. xiv + 574, with 746 illustrations. *(Postage 1s. 4d., abroad 2s. 8d.)* 76s. 6d.

**A HANDBOOK OF SURGERY**

By R. C. B. LEDLIE, M.B., B.S., F.R.C.S., and M. H. HARMER, M.B., B.CHIR., F.R.C.S. "Well-arranged and up-to-date . . . the writing is clear and the illustrations all serve a necessary purpose. The book will prove a real boon."—*Practitioner.*
Pp. viii + 536, with 56 illustrations. *(Postage 1s., abroad 2s.)* 21s. 0d.

**BAILLIÈRE, TINDALL AND COX LTD.**

**7-8, Henrietta Street, London, W.C.2**

